# Investigation of Phospholipid Differences in Valproic Acid-Induced Autistic Mouse Model Brain Using Mass Spectrometry Imaging

**DOI:** 10.3390/metabo13020178

**Published:** 2023-01-25

**Authors:** Hyun Jun Jang, Kyoung Ja Kwon, Chan Young Shin, Ga Seul Lee, Jeong Hee Moon, Tae Geol Lee, Sohee Yoon

**Affiliations:** 1 Center for Cognition and Sociality, Institute for Basic Science, 55 Expo-ro, Yuseong-gu, Daejeon 34126, Republic of Korea; 2 Department of Medical Science, School of Medicine, Konkuk University, 120 Neungdong-ro, Gwangjin-gu, Seoul 05029, Republic of Korea; 3 Department of Pharmacology, School of Medicine, Konkuk University, 120 Neungdong-ro, Gwangjin-gu, Seoul 05029, Republic of Korea; 4 Core Research Facility & Analysis Center, Korea Research Institute of Bioscience and Biotechnology (KRIBB), 125 Gwahak-ro, Yuseong-gu, Daejeon 34141, Republic of Korea; 5 Bio-Imaging Team, Safety Measurement Institute, Korea Research Institute of Standards and Science (KRISS), 267 Gajeong-ro, Yuseong-gu, Daejeon 34113, Republic of Korea

**Keywords:** phospholipid, autism spectrum disorder, valproic acid, MALDI, mass spectrometry imaging, mouse brain

## Abstract

Autism is a neurodevelopmental disorder for which the cause and treatment have yet not been determined. The polyunsaturated fatty acid (PUFA) levels change rapidly in the blood or cerebrospinal fluid of autistic children and PUFAs are closely related to autism spectrum disorder (ASD). This finding suggests that changes in lipid metabolism are associated with ASD and result in an altered distribution of phospholipids in cell membranes. To further understand ASD, it is necessary to analyze phospholipids in organs consisting of nerve cells, such as the brain. In this study, we investigated the phospholipid distribution in the brain tissue of valproic acid-induced autistic mice using matrix-assisted laser desorption/ionization mass spectrometry imaging (MALDI-MSI). Phospholipids including phosphatidylcholine, phosphatidylethanolamine, and phosphatidylserine were identified in each brain region and exhibited differences between the ASD and control groups. These phospholipids contain docosahexaenoic acid and arachidonic acid, which are important PUFAs for cell signaling and brain growth. We expect that the differences in phospholipids identified in the brain tissue of the ASD model with MALDI-MSI, in conjunction with conventional biological fluid analysis, will help to better understand changes in lipid metabolism in ASD.

## 1. Introduction

Mass spectrometry imaging (MSI) provides information about the spatial distribution and chemical composition of various molecules on a sample surface by irradiating the surface with an ionized beam and recording ion signals at each position [1]. Matrix-assisted laser desorption/ionization (MALDI) is a widely used ionization method for the MSI of biomolecules in living tissues. It is a soft ionization technique that can generate intact molecular ions of labile biomolecules such as proteins, DNA, and lipids [2]. Therefore, MALDI-MSI has been widely used to study disease biomarkers for which location information of disease-specific biomolecules is important [3,4,5]. Currently, biomarker research using MSI is actively being conducted to determine where disease-related biomolecules are located within the brain for degenerative diseases such as Alzheimer’s disease, Parkinson’s disease, Huntington’s disease, multiple sclerosis, and amyotrophic lateral sclerosis [6,7].

Another degenerative disease, autism spectrum disorder (ASD), is a complex neurodevelopmental disorder characterized by deficits in social communication and interaction because of a variety of genetic and environmental factors [8,9]. ASD is accompanied by various comorbid symptoms such as attention-deficit/hyperactivity disorder, anxiety, seizures, hyperactivity, sensory hyper- or hypo-activation, and intellectual disability. Therefore, ASD onset disrupts overall brain function [10]. Research to identify the biological causes of ASD and discover biomarkers is focusing on genes and expressed proteins involved in synaptic function [10,11]. However, imaging studies that show differences in the distribution of biochemical markers according to brain region are rare. Therefore, MALDI-MSI may be an optimal approach.

Neuroinflammation is considered a major cause of cellular damage in autistic children and is characterized by brain cell activation and increased cytokine production [12]. When neuroinflammation occurs, inflammatory cytokine production increases, resulting in changes in the activity of phospholipase A2, which is a target protein of inflammation. Changes in phospholipase A2 activity lead to altered phospholipid expression in neuronal membranes and neurons [13]. According to several studies, differences in the fatty acids of phospholipids present in the red blood cell membrane were found in autistic children [14]. Additional differences were found in the amount of polyunsaturated fatty acids (PUFAs) such as docosahexaenoic acid (DHA) and arachidonic acid (AA) [14,15]. The cytosolic phospholipase A2 concentration was significantly increased and a decreased phospholipid level was observed in the serum plasma and cerebrospinal fluid of autistic children [16]. Taken together, these results suggest that ASD pathogenesis and changes in phospholipid metabolism are closely related.

Valproic acid (VPA)-exposed animals comprise one of the most widely used ASD models [17,18,19,20]. The offspring of female rats exposed to VPA during pregnancy develop brain damage and exhibit autistic symptoms with prolonged repetitive behaviors compared with controls [18]. In this study, using MALDI-MSI, we investigated the differences in phospholipid distribution in the brain tissue of mice with autism induced by VPA exposure during pregnancy. In addition, by examining differences in phospholipids, we indirectly confirmed alterations in PUFAs which are derived from dissociation from phospholipids. Differences between the control group and the ASD group caused by differences in phospholipid distribution were compared by multivariate analysis.

## 2. Materials and Methods

### 2.1. VPA-Induced ASD Mouse Model and Sample Preparation for MALDI-MSI

Construction of the ASD mouse model and acquisition of brain tissue samples were all performed at Konkuk University. All experimental procedures were performed following approval by the institutional animal care and use committee of Konkuk University (KU19209). A 10 mg/mL solution of VPA prepared in 0.9% saline was injected into pregnant ICR mice (300 mg/kg of body weight) on embryonic day 10. For the control group, only 0.9% saline was administered to pregnant mice. Hyperactivity and lack of sociability, which are symptoms of ASD, were confirmed through an open field test and a social interaction test conducted approximately 3 weeks after birth. The brain tissues from these mouse pups were sectioned and used for MSI.

The dissected brain tissue was immediately stored at −80 °C until use. The frozen mouse brain was cut into 12 μm thick sagittal sections using a Leica CM3050 cryostat maintained at −20 °C (Leica Biosystems, IL, USA). The tissue used for measurement was randomly selected from the left hippocampus. The tissue sections were thaw-mounted onto ITO glass slides (Bruker Daltonik GmbH, Bremen, Germany), and the sample slides were vacuum dried in a vacuum pump for 2 h. 

**Figure 1:**
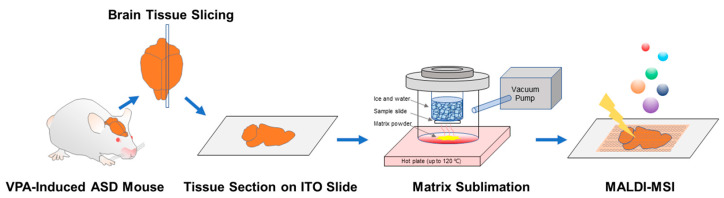
Matrix-assisted laser desorption/ionization mass spectrometry imaging (MALDI-MSI) workflow for autism spectrum disorder (ASD) mouse brain tissues. The brains of valproic acid-induced ASD mice were removed, and the brain tissue was cut to a thickness of 12 µm and attached to an ITO slide by thaw-mounting. After sufficiently drying the tissue sections, 1,5-diaminonaphthalene was sublimated on the sample. MALDI-MSI was performed by irradiating whole tissue sections with a laser beam focused at 50 µm and recording the ion signals produced.

To perform MALDI-MSI, 1,5-diaminonaphthalene (1,5-DAN), a matrix optimized for phospholipid detection, was applied to dried tissue sections [21]. Matrix deposition was performed using a custom-made sublimation apparatus composed of a top and bottom quartz glass chamber, an O-ring, and a clamp. The upper and lower glass chamber had bottom diameters of 80 mm and 120 mm, respectively, and the bottom part was flat so that the sample plate and matrix could be placed on it. First, 10 mg of 1,5-DAN powder (Sigma-Aldrich, MO, USA) was placed on the bottom of the lower glass chamber, and the sample slide was fixed to the upper glass chamber using tape, as shown in Figure 1. The chamber was clamped, and a vacuum was allowed to reach approximately 7 mbar. After approximately 5 min, ice and water were added to the upper glass chamber and a hot plate was heated to 120 °C. When the matrix powder was completely sublimated, the hot plate was turned off and the apparatus was cooled to room temperature. Protective equipment was worn during the entire sublimation process and the work was performed under a hood.

### 2.2. MALDI-MSI

MALDI-MSI measurements of the brain section samples were performed using a rapifleX MALDI Tissuetyper (Bruker Daltonik GmbH) equipped with a smartbeam 3D laser (Nd:YAG 355 nm) operating at a repetition rate of 10 kHz. Mass spectra were recorded for 50 laser shots in both the positive and negative ion modes. The mass range was set to a mass-to-charge ratio (*m*/*z*) of 400–1200, and the laser focusing size was set to 50 μm. Cesium iodide was used to calibrate the *m*/*z* range in both ion acquisition modes. MALDI mass image spectra were visualized using FlexImaging 5.0 software (Bruker Daltonik GmbH).

### 2.3. Tandem Mass Spectrometry

To identify phospholipid species, tandem mass spectrometry (MS) was performed using lipid extracts obtained from the brain tissue, as described in a previous study [22]. The lipid extract was analyzed using an Orbitrap Elite Hybrid Ion Trap-Orbitrap mass spectrometer (Thermo Scientific, MA, USA). The lipid extract was then electrosprayed at a flow rate of 3 μL/min through a stainless-steel needle (gauge 32) and the ion transfer tube was maintained at 320 °C. The source voltages were 4.0 kV and −3.2 kV in the positive and negative ion modes, respectively. The sheath gas flow rate was 7 arbitrary units and the auxiliary gas and sweep gas flow rates were 2 arbitrary units. Phospholipid ions were dissociated in a higher-energy collisional dissociation collision cell and analyzed using an Orbitrap analyzer. The microscan count was set to 1 and the maximum ion injection time was set to 200 ms in the positive ion mode and 500 ms in the negative ion mode. The normalized collision energy was set to 20–50 in the positive ion mode and 25–110 in the negative ion mode; the isolation width was set from 0.6–1.0. All MS and tandem MS spectra were obtained with a mass resolution of 60,000 at an *m*/*z* of 400 using Tune Plus 2.7 SP2 software (Thermo Scientific). The classification and structure of the lipid molecules were based on LIPID MAP (https://www.lipidmaps.org, accessed on 26 November 2023) and MassBank of North America (https://mona.fiehnlab.ucdavis.edu/, accessed on 26 November 2023).

### 2.4. Data Analysis

MSI data were analyzed using SCiLS Lab 2019a Pro software (Bruker Daltonik GmbH). Imported data were normalized to the total ion count and the *m*/*z* interval width was set to ±0.2–0.4 Da. The number of manually selected peaks was 71 in the positive ion mode and 89 in the negative ion mode. Principal component analysis (PCA) supported by SCiLS software was used as a multivariate analytic tool to compare differences between the groups. Individual spectra from control and VPA-induced ASD mice were used for the PCA. Five principal components were obtained, and the three principal components with the largest difference were used for the three-dimensional score plot. 

## 3. Results and Discussion

### 3.1. Brain Compartmentalization of the ASD Mouse Model

**Figure 2:**
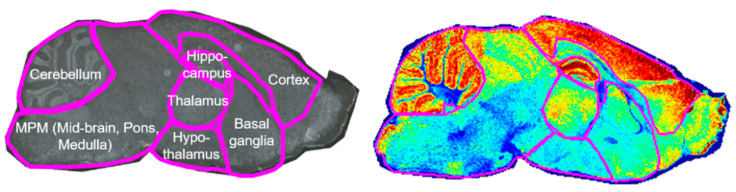
Mouse brain compartmentalization used in this study. The sagittal plane of the mouse brain was divided into seven regions: cerebellum; hippocampus; cortex; thalamus; hypothalamus; midbrain, pons, and medulla; and basal ganglia (left image). The right image represents a whole-brain section mass spectrometry image of [PE(16:0/22:6)−H]^−^ divided into seven regions.

The brain is a complex organ composed of several regions that perform various functions such as cognition, emotion, learning, and behavior. The brain regions that perform these functions have well-characterized anatomical divisions. Therefore, rather than analyzing the entire brain at once, separately observing changes occurring in each region may be more helpful for understanding brain diseases. Previous studies of ASD have also divided the brain into different regions to investigate neuronal damage, connectivity deficits between brain functions, and morphological alterations in each region including the cerebellum [23,24]; cortex [25,26]; hippocampus [27,28]; thalamus [29,30]; hypothalamus [31]; midbrain, pons, and medulla (MPM) [32]; and basal ganglia [33,34,35]. In this study, compared with the control group, the differences in phospholipids caused by ASD in seven brain regions that could be distinguished with MALDI-MSI were examined. The MALDI-MSI results of a mouse brain segmented into seven compartments are shown in Figure 2.

### 3.2. Changes in Phospholipid Levels in the VPA-Induced ASD Mouse Brain

**Figure 3:**
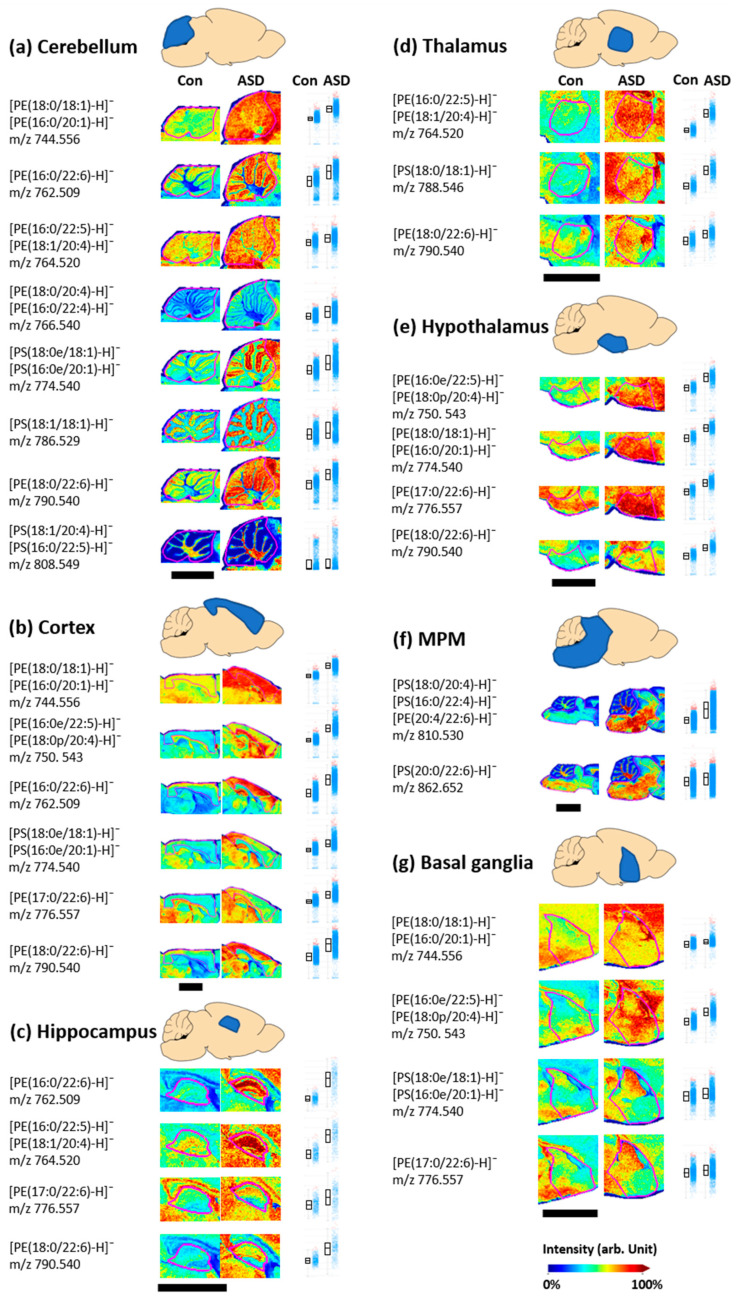
Matrix-assisted laser desorption/ionization mass spectrometry imaging (MALDI-MSI) results and intensity box plots of negatively charged phospholipid ions detected in the control (Con) and valproic acid-induced autism spectrum disorder model (ASD) groups. Comparison of the relative intensity of negatively charged phospholipids was performed in the cerebellum (**a**), cortex (**b**), hippocampus (**c**), thalamus (**d**), hypothalamus (**e**), MPM (**f**), and basal ganglia (**g**), respectively. The magenta lines shown on the MALDI-MSI results indicate each part of the brain tissue, and the scale bars are 3 mm. In the intensity box plots, the *y*-axis represents the total ion count normalized intensity, and the horizontal line of the box part represents the median intensity defined so that the number of spectra with low and high values are the same. The spectra for which the intensities of a specific *m*/*z* value are between the lower and upper quantiles are shown by blue dots. Spectra with intensities outside of these intensity intervals are shown by red dots.

Phospholipids that exist as positively and negatively charged ions in vivo can be detected in the positive and negative ion modes of MALDI-MS. We performed MALDI-MSI to identify the phospholipids present in brain tissue by measuring ions of both polarities in the same tissue section. Figure 3 shows the distribution in the brain and the relative intensity of the negatively charged phospholipid ion peaks that were different between the control and ASD groups. In the MALDI-MSI negative ion mode results, 31 lipid peaks in seven brain regions showed differences between control and ASD tissues as shown in Figure 3. Information including the *m*/*z* value, ion types, and fatty acid types of phospholipids is shown in Table 1.

In the cerebellum of ASD tissue, eight lipid ions, five phosphatidylethanolamine (PE) ions, and three phosphatidylserine (PS) ions showed higher intensities than those in the control tissue (Figure 3a)—PE(18:0/18:1, 16:0/20:1) at *m*/*z* 744.556, PE(16:0/22:6) at *m*/*z* 762.509, PE(16:0/22:5, 18:1/20:4) at *m*/*z* 764.520, PE(18:0/20:4, 16:0/22:4) at *m*/*z* 766.540, PS(18:0e/18:1, 16:0e/20:1) at *m*/*z* 774.540, PS(18:1/18:1) at *m*/*z* 786.529, PE(18:0/22:6) at *m*/*z* 790.540, and PS(18:1/20:4, 16:0/22:5) at *m*/*z* 808.549. In addition, six lipid ions (PE(18:0/18:1 and/or 16:0/20:1) at *m*/*z* 744.556, PE(16:0e/22:5, 18:0p/20:4) at *m*/*z* 750.543, PE(16:0/22:6) at *m*/*z* 762.509, PS(18:0e/18:1, 16:0e/20:1) at *m*/*z* 774.540, PE(17:0/22:6) at *m*/*z* 776.557, and PE(18:0/22:6) at *m*/*z* 790.540) in the cortex (Figure 3b), four lipid ions (PE(16:0/22:6) at *m*/*z* 762.509, PE(16:0/22:5, 18:1/20:4) at *m*/*z* 764.520, PE(17:0/22:6) at *m*/*z* 776.557, and PE(18:0/22:6) at *m*/*z* 790.540) in the hippocampal region (Figure 3c), three lipid ions (PE(16:0/22:5, 18:1/20:4) at *m*/*z* 764.520, PS(18:0/18:1) at *m*/*z* 788.546, and PE(18:0/22:6) at *m*/*z* 790.540) in the thalamus (Figure 3d), four lipid ions (PE(16:0e/22:5, 18:0p/20:4) at *m*/*z* 750.543, PE(18:0/18:1, 6:0/20:1) at *m*/*z* 774.540, PE(17:0/22:6) at *m*/*z* 776.557, and PE(18:0/22:6) at *m*/*z* 790.540) in the hypothalamus (Figure 3e), two lipid ions (PS(18:0/20:4, 16:0/22:4, 20:4/22:6) at *m*/*z* 810.530 and PS(20:0/22:6) at *m*/*z* 862.652) in the MPM region (Figure 3f), and four lipid ions (PE(18:0/18:1, 16:0/20:1) at *m*/*z* 744.556, PE(16:0e/22:5, 18:0p/20:4) at *m*/*z* 750.543, PS(18:0e/18:1, 16:0e/20:1) at *m*/*z* 774.540, and PE(17:0/22:6) at *m*/*z* 776.557) in the basal ganglia (Figure 3g) exhibited higher intensity in ASD tissue than in control tissue. 

Some phospholipid ions showed relative intensity differences in several regions. PE(18:0/22:6) showed a higher intensity in the ASD tissue than in the control tissue in five regions including the cerebellum, cortex, hippocampus, thalamus, and hypothalamus. In addition, PE(18:0/18:1, 16:0/20:1) in four regions (cerebellum, cortex, hypothalamus, and basal ganglia) and PE(17:0/22:6) in four regions (cortex, hippocampus, hypothalamus, and basal ganglia) showed a higher intensity in the ASD tissue than in the control tissue. 

Several studies have shown that ASD causes an imbalance in phospholipase regulation, resulting in changes in the expression and concentration distribution of phospholipids present in brain cell membranes [16]. Several studies investigating changes in biomolecule concentrations in the body fluids of autistic children reported differences in phospholipid levels, such as decreased PE and PS molecule concentrations in the blood and increased PE molecule concentrations, including PE(16:0/22:6), in the plasma [36]. All of these reports indicated that the expression and distribution of phospholipids are altered by the onset of ASD. Our MALDI-MSI results also showed that the PE molecule distributions had stronger intensity in the brain tissue of the ASD group than that of the control group, showing a similar trend to that of biological fluid sample studies [36].

Phosphatidylcholine (PC) is the most abundant phospholipid in cells and has been linked to several biological processes including intracellular cholesterol transport and membrane cholesterol homeostasis [37]. Lysophosphatidylcholine (LPC) is mainly produced by PC turnover via phospholipase A2 and is associated with neurodegenerative diseases [38]. MALDI-MSI of positively charged phospholipids in autistic brain tissue showed that PC and LPC molecules exhibited different distributions than those in the control group.

**Figure 4:**
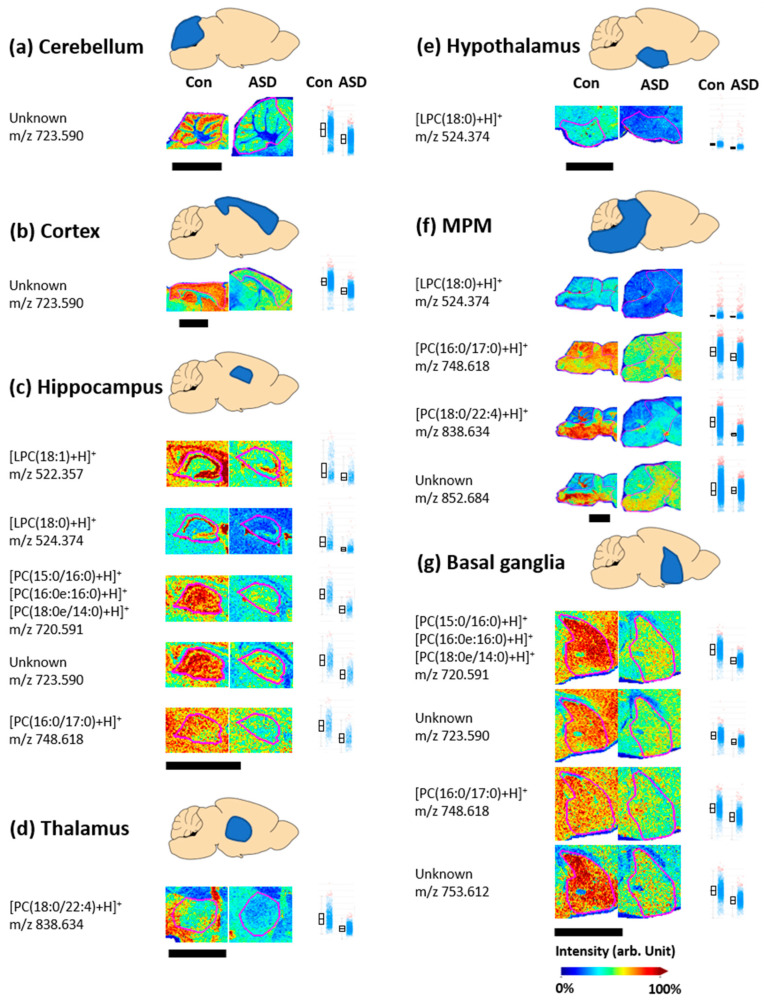
Matrix-assisted laser desorption/ionization mass spectrometry imaging (MALDI-MSI) results and intensity box plots of positively charged phospholipid ions detected in the control (Con) and valproic acid-induced autism spectrum disorder model (ASD) groups. Comparison of the relative intensity of positively charged phospholipids was performed in the cerebellum (**a**), cortex (**b**), hippocampus (**c**), thalamus (**d**), hypothalamus (**e**), MPM (**f**), and basal ganglia (**g**), respectively. The magenta lines shown in the MALDI results indicate each part of the brain tissue and the scale bars are 3 mm. In the intensity box plots, the *y*-axis represents the total ion count normalized intensity, and the horizontal line of the box part represents the median intensity defined so that the number of spectra with low and high values are the same. The spectra for which the intensities of specific *m*/*z* values are between the lower and upper quantiles are shown by blue dots. Spectra with intensities outside of these intensity intervals are shown by red dots.

In the positive ion mode, 18 lipid peaks and six unknown ions in seven regions showed differences in the control and ASD tissues, as shown in Figure 4. In the cerebellum and cortex regions, unknown ions (*m*/*z* 723.590) showed higher intensity in the control tissue than in the ASD tissue (Figure 4a,b). In addition, four lipid ions (LPC(18:1) at *m*/*z* 522.357, LPC(18:0) at *m*/*z* 524.374, PC(15:0/16:0, 16:0e:16:0, 18:0e/14:0) at *m*/*z* 720.591, and PC(16:0/17:0) at *m*/*z* 748.618) and one unknown ion (*m*/*z* 723.590) in the hippocampus (Figure 4c); one lipid ion (PC(18:0/22:4) at *m*/*z* 838.634) in the thalamus (Figure 4d); one lipid ion (LPC(18:0) at *m*/*z* 524.374) in the hypothalamus (Figure 4e); three lipid ions (LPC(18:0) at *m*/*z* 524.374, PC(16:0/17:0) at *m*/*z* 748.618, and PC(18:0/22:4) at *m*/*z* 838.634), and one unknown ion (*m*/*z* 852.684) in the MPM region (Figure 4f); and two lipid ions (PC(15:0/16:0 and/or 16:0e:16:0 and/or 18:0e/14:0) at *m*/*z* 720.591, and PC(16:0/17:0) at *m*/*z* 748.618) and two unknown ions (*m*/*z* 723.590 and *m*/*z* 753.612) in the basal ganglia (Figure 4g) had a higher intensity in the control tissue than in the ASD tissue.

The decreases in PC and LPC reported in the present study are supported by previous study results [39,40,41,42]. Choline plays an important role as a methyl group donor in the synthesis of PC, which is considered an essential component of membrane phospholipids [16,43]. Choline levels in the plasma of autistic children have been reported to be lower than those of healthy controls. This low choline level is consistent with the MALDI-MSI results that showed lower PC levels in ASD tissue. 

The neural membranes in the brain are rich in lipids such as major PC, PE, PS, PI, sphingolipid, and cholesterol molecules and have specific PUFA levels [44,45] Phospholipids serve not only as structural components of cell membranes but also as precursors for various secondary messengers such as AA, DHA, 1,2-diacylglycerol, and phosphatidic acid [16]. The high PLA2 activity found in red blood cells of degenerative autistic children affects the hydrolysis of neuronal membrane phospholipids and increases the rate of loss of PUFAs such as AA and DHA, thereby altering brain phospholipid metabolism. Altered lipid metabolism exacerbates functional and structural changes in cell membrane phospholipids and contributes to central nervous system damage [16,46].

Phospholipids that showed significant differences in autistic brain tissue in the MALDI-MSI results included PUFAs. DHA and AA are the major PUFAs that are abundantly distributed in vertebrate brain neurons [46]. DHA is involved in cell signaling and has an important structural role in the brain. AA is crucial for brain growth [46,47,48]. Abnormalities in brain neurons caused by oxidative stress or inflammation excessively activate PLA2 and promote the hydrolysis of sn2 bonds in phospholipids [49]. Accordingly, AA(20:4) and DHA(22:6) are released, resulting in changes in PUFA levels. Changes in the levels of PUFAs, which are highly susceptible to damage from oxidative stress, are associated with ASD [49]. Our MSI results showed differences in phospholipids containing PUFAs according to brain regions, consistent with previous studies showing that the PUFA distribution was altered in ASD. Among the phospholipids exhibiting altered levels in autistic brain tissue, many phospholipids included DHA(22:6) and AA(20:4), as shown in Figure 3. DHA(22:6) is an unsaturated fatty acid that binds to the sn2 position of PE(16:0/22:6), PE(17:0/22:6), PE(18:0/22:6), PE(20:4/22:6), and PS(20:0/22:6). In addition AA(20:4) can be found in the side chain of the sn2 position of PE(18:1/20:4), PE(18:0/20:4), PE(18:0p/20:4), and PS(18:1/20:4). These phospholipids were distributed at increased concentrations throughout the brain of the ASD mouse model, suggesting that abnormalities in brain cells lead to changes in PUFA levels. Although the patterns of differences in the concentrations of negatively charged phospholipids in autistic patients are slightly different in several studies, overall, this finding explains the changes in phospholipid levels caused by PUFA release when ASD is induced [16,36,42].

The decreased PC levels in the ASD group shown by MALDI-MSI can be explained in conjunction with choline, the head group of this phospholipid. Choline is involved in the synthesis of PC, which is an essential membrane phospholipid component and provides a methyl group in the synthesis of the neurotransmitter acetylcholine [43]. Changes in choline metabolism can cause abnormalities in PC and neurotransmitter synthesis, leading to a disruption of cell membrane function and neurotransmitter systems [50]. Another mouse model in which ASD was induced by the injection of propionic and butyric acid showed the altered metabolism of phospholipids, including PC, and impaired language function [51]. 

Although fatty acid distributions were not directly measured in this study, MALDI-MSI of the brain tissue indirectly demonstrated that PUFA levels were altered when ASD was induced through changes in the phospholipid distribution. This study, which investigated phospholipid differences in the brain tissue of a disease model, indicated that metabolic changes in various biological tissues can be studied through MSI. MALDI-MSI can be used to discover biomarkers through comparative analysis of biomolecules in brain diseases or diseases in which the location information of biological materials is important. Few studies have investigated metabolic changes in the brain tissue in ASD models using MSI. Therefore, this technique can provide complementary information for biomarker discovery by examining the distribution of biochemical molecules that show specific changes in ASD.

### 3.3. Multivariate Analysis

We used PCA as a multivariate analysis method to compare differences between groups. Because phospholipids are classified into different types according to a difference of 2 Da in the mass spectrum, it is difficult to automatically distinguish between lipid ion peaks and their isotopic peaks using software packages. Therefore, to avoid the overlapping of lipid ion peaks and isotope peaks, monoisotopic peaks of phospholipids were manually selected and PCA was performed. In the three-dimensional score plot of the seven brain regions, the lipid peaks detected in negative and positive ion modes displayed well-distinguished differences between VPA-induced ASD mice and control mice.

**Figure 5:**
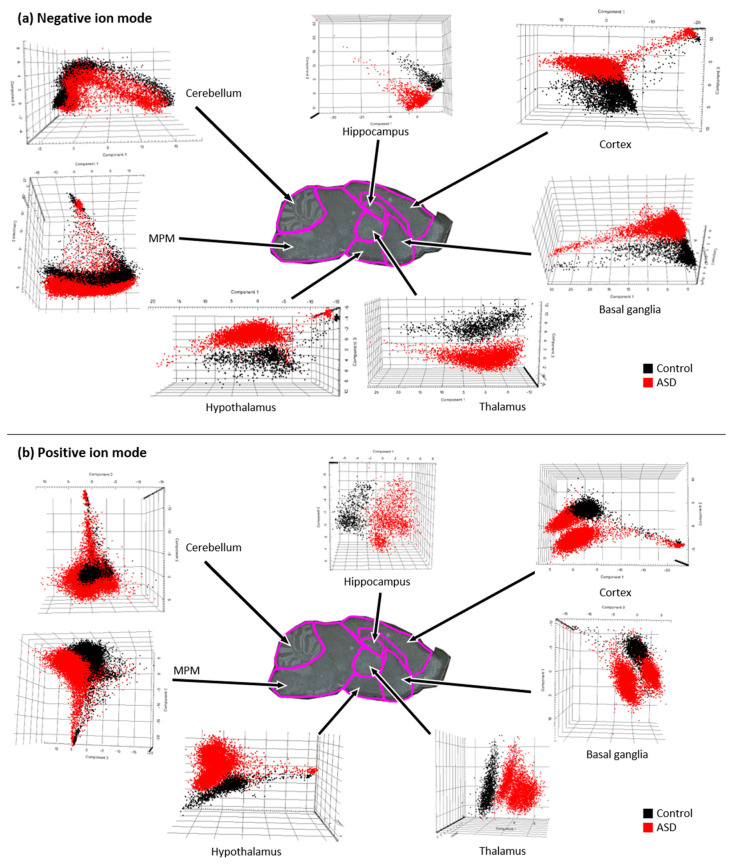
Three−dimensional principal component analysis score plots from seven brain regions in valproic acid-induced autism spectrum disorder (ASD) and control mice. Three−dimensional plots show three component scores obtained from individual spectra of negative ions (**a**) and positive ions (**b**) measured in seven brain regions.

The PCA results shown in Figure 5 indicate differences in brain phospholipids between the ASD and control groups. In terms of brain regions, the hippocampus, hypothalamus, and thalamus showed marked differences between the ASD and control groups in both ion acquisition modes. In the positive ion mode, the cerebellum, MPM, cortex, and basal ganglia showed differences between the two groups, but these differences were less distinct than those in the hippocampus, hypothalamus, and thalamus (Figure 5b). However, in the negative ion mode, significant differences were observed between the two groups in all seven brain regions, which were clearly distinguished (Figure 5a). Regarding phospholipid types, the PE and PS ions in the negatively charged state in ASD brain tissue discovered by MALDI-MSI were disease-specific phospholipids that were distinct from those in the control group.

## 4. Conclusions

In this study, we measured the distribution of phospholipids in brain tissue using the MALDI-MSI technique in a VPA-induced autism model. The differences in phospholipid ion levels and distributions in the autism group compared with the control group could be presented as images by brain region. In addition, differences in ion signals of phospholipids containing PUFAs, which were presumed to be potential autism biomarkers in previous studies, were also identified. These MSI results reflect changes in lipid metabolism that occur when autism develops. In this study, disease-specific phospholipids were visualized as images by applying biomarker discovery techniques that were previously conducted in biological fluids of children with autism to brain tissue. The addition of conventional biochemical staining analysis such as immunohistochemical or Western blot to this MSI study will enable future research to identify biological mechanisms that can explain changes in phospholipid levels in the brain of autism. MSI-based autism model research may be useful to understand autism, for which the cause and treatment have not been identified, and to discover biomarkers for diagnosis.

## Figures and Tables

**Table 1:** Identification of phospholipid ions detected in brain tissue of control and valproic acid-induced autism mice. PUFAs are marked in red.

	*m*/*z*	Phospholipid Ions	Fatty Acids	Fragment Ions	References
Positive ions	522.3571	[LPC + H]^+^	18:1	184, 240, 258, 504	Lipid DB ^a^
	524.3729	[LPC + H]^+^	18:0	86, 104, 109, 125, 163, 184, 258, 341, 447, 506	Lipid DB ^a^
	720.5916	[PC + H]^+^	15:0/16:0 16:0e/16:0 18:0e/14:0	184, 537 184, 537 184, 482, 496	Lipid DB ^a^
	723.5899	Unknown			
	748.5872	[PC + H]^+^	16:0/17:0	184, 478, 492, 496, 510	Lipid DB ^a^
	753.6016	Unknown			
	838.6337	[PC + H]^+^	18:0/22:4	184, 506, 524	Lipid DB ^a^
	852.6444	Unknown			
Negative ions	744.5547	[PE − H]^−^	18:0/18:1 16:0/20:1	79, 140, 153, 281, 283, 460, 462, 478, 480 79, 140, 153, 255, 309, 452, 488, 506	Lipid DB ^b^
	750.5442	[PE − H]^−^	16:0e/22:5 18:0p/20:4	79, 153, 258, 329, 420, 438 79, 153, 259, 303, 446, 464	Lipid DB ^b^
	762.5082	[PE − H]^−^	16:0/22:6	79, 97, 122, 140, 153, 255, 283, 327, 434, 452, 506, 524	Lipid DB ^b^
	764.5237	[PE − H]^−^	16:0/22:5 18:1/20:4	79, 97, 122, 140, 153, 255, 285, 329, 434, 452 79, 97, 122, 140, 153, 259, 281, 303, 460, 478, 482, 500	Lipid DB ^b^
	766.5393	[PE − H]^−^	18:0/20:4 16:0/22:4	79, 97, 122, 140, 153, 259, 283, 303, 462, 480, 500 79, 97, 122, 140, 153, 255, 331, 434, 452	Lipid DB ^b^
	774.5392	[PS − H]^−^	18:0e/18:1 16:0e/20:1	79, 153, 281, 405, 423, 687 79, 153, 309, 377, 687	Lipid DB ^b^
	776.5565	[PE − H]^−^	17:0/22:6	79, 140, 153, 269, 283, 327, 448, 466	Lipid DB ^b^
	786.5299	[PS − H]^−^	18:1/18:1	79, 97, 153, 281, 417, 435, 699	Lipid DB ^b^
	788.5458	[PS − H]^−^	18:0/18:1	79, 97, 153, 281, 283, 435, 437, 701	Lipid DB ^b^
	790.5398	[PE − H]^−^	18:0/22:6	79, 97, 122, 140, 153, 283, 327, 462, 480, 506, 524	Lipid DB ^b^
	808.5107	[PS − H]^−^	18:1/20:4 16:0/22:5	79, 97, 153, 259, 281, 303, 417, 435, 439, 721 79, 97, 153, 255, 329, 391, 721	Lipid DB ^b^
	810.5300	[PE − H]^−^ [PS − H]^−^	18:0/20:4 16:0/22:4 20:4/22:6	79, 97, 153, 259, 283, 303, 419, 437, 439, 457, 723 79, 97, 153, 255, 331, 391, 467, 723 79, 97, 153, 259, 283, 303, 327	Lipid DB ^b^
	862.6080	[PS − H]^−^	20:0/22:6	79, 97, 153, 283, 311, 327, 447, 465, 775	Lipid DB ^b^

Lipid DB ^a^: MassBank of North America, Lipid DB ^b^: LIPID MAPS.

## Data Availability

Data is not publicly available due to privacy.
